# Recycling PVC with scCO_2_: From Soft to Rigid PVC

**DOI:** 10.1021/acssuschemeng.4c03743

**Published:** 2024-08-28

**Authors:** Frederique A. Versteeg, Diana A. W. M. Bollen, Francesco Picchioni

**Affiliations:** Department of Chemical Engineering – Product Technology, University of Groningen, Nijenborgh 4, 9747 AG Groningen, The Netherlands

**Keywords:** scCO_2_, extraction, plasticizers, phthalates, PVC, Sovová

## Abstract

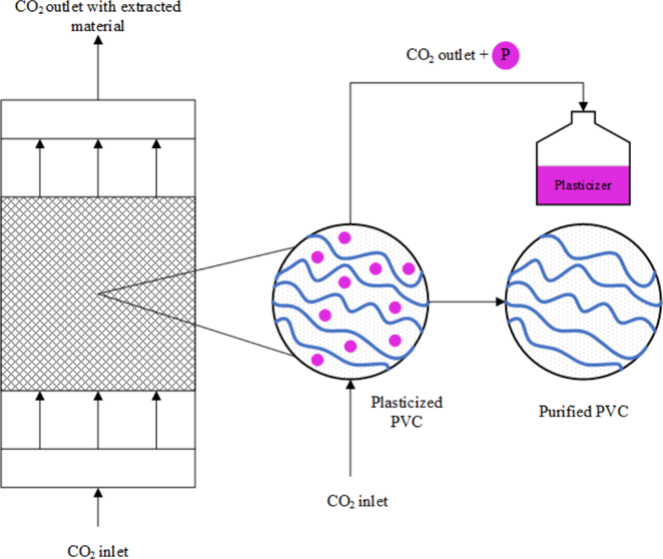

This study explores using extraction with supercritical carbon dioxide to remove plasticizers from poly(vinyl chloride) (PVC), with the aim to enhance recycling processes. Experiments were performed at 100 to 500 bar with temperatures varying from 75 to 110 °C. The results show that continuous supercritical carbon dioxide efficiently removes bis(2-ethylhexyl) phthalate (DOP) from PVC with extraction efficiencies of more than 98% which resulted in PVC samples with over 99.5% purity. A process model based on the Sovová model accurately describes the extraction process. The impact of the extraction process on polymer properties and associated molecular structures like viscosity, morphology, and mechanical properties was also evaluated using gel permeation chromatography, rheology, tensile test, and scanning electron microscopy.

## Abstract 2

This study utilizes scCO_2_ extraction as a green technology for toxic phthalates removal from plasticized PVC and analyzes its effect on the PVC properties to enable sustainable recycling of industrial waste PVC.

## 1 Introduction

Poly(vinyl chloride) (PVC) is one of the most frequently used plastics worldwide. Unlike elastomers, thermoplastics are usually not formulated with a large number of additives. However, PVC is an exception because of its remarkable rigidity at ambient temperatures. Therefore, PVC is combined with a range of special additives such as thermal stabilizers, plasticizers, mineral fillers, etc. This makes PVC a versatile polymer, which possesses many different properties, and can be used in a very wide variety of applications.^1−3^ However, PVC waste management is often complicated due to the presence of all of these additives that diminish the possibility of PVC recycling.^4,5^

When the focus is on recycling, two types of PVC can be distinguished, namely, rigid and soft/flexible PVC. Rigid PVC is mainly used in piping, nonfood packaging, and plastic (bank) cards. By adding plasticizers, the PVC can be made more flexible and used as flooring, cable insulation, and factually as an alternative for rubber applications. However, plasticizers do not form a chemical bond with the polymer. Therefore, they can be leached out from the end-product to the package or, ultimately, the environment. While rigid PVC can be mechanically recycled, soft PVC is landfilled due to the high content of phthalates.^4,5^ Therefore, it is interesting to create rigid PVC from soft PVC because unplasticized PVC with proper thermal stabilizers can be processed multiple times without degradation.^3,6^

The concentration of plasticizers in a typical PVC formulation varies between 15 and 50 wt % depending on the final product requirements.^7^ The most commonly used plasticizers in PVC are phthalates, which are esters of phthalic acid. Phthalates make up 80% of the total volume of plasticizers used today. Di-2-ethylhexyl phthalate (DOP), diisononyl phthalate (DINP), diisodecyl phthalate (DIDP), and 2-propyl heptyl phthalate (DPHP) are the four most frequently used phthalates.^2^ Their use in PVC is of concern because phthalates are linked to endocrine modulation, particularly to toxic effects on reproduction and fertility.^8,9^ Moreover, there are very strict regulations and costs involved, considering the presence of phthalates in processes. However, it should be noted that for a limited amount of PVC-based products, it is allowed that there is still, e.g., DOP present before they are further processed/upgraded. This allowance is subject to strict legal regulations. Generally, it is forbidden to process this class of PVC materials without these special permits. The consequence is that a strict control beforehand is required to classify the streams in order to prevent mixed streams from being processed. This makes recycling even more difficult because recyclers deal with waste generated by others, including the waste from manufacturers who are authorized to use these plasticizers such as medical product producers.^10^ Hence, it makes sense to introduce a preliminary step in upcoming recycling processes involving the extraction of phthalates.

A relatively novel idea that could aid in the removal of additives or impurities from plastics is supercritical fluid extraction (SFE). The technique is used to separate via extraction one component from another by using a supercritical fluid. A fluid is in a supercritical state when the pressure and temperature are above its critical point. The diffusivity and gas-like viscosity, almost no surface tension, liquid-like density, and pressure-dependent solvating power result in improved extraction capabilities.^11^ One of the most commonly used supercritical fluids is carbon dioxide (CO_2_). Supercritical carbon dioxide (scCO_2_) is regarded as a green solvent that can be conveniently used due to its mild critical conditions (304 K and 7.38 MPa), nonflammability, nontoxicity, low cost, and relative abundance.^12^

A lot of research is available for fast analyzing methods for plasticizers in PVC with the use of scCO_2_ instead of removing it. Hunt et al. focused on optimizing extraction conditions for the extraction of PVC with scCO_2_.^13^ The PVC contained diisooctyl phthalate (DIOP), chlorinate polythene wax, and trace levels of Topanol CA. They determined that at 95 °C and 450 bar for 30 min 97.3% of liquid extractable material was extracted by SFE with excellent reproducibility. Marín et al. also developed an analytical method in order to determine the concentrations of dibutyl phthalate (DBP) and DOP in PVC.^14^ They prepared the plastisols by mixing powdered PVC with the selected plasticizers. It appeared that the use of scCO_2_ led to extraction efficiencies of more than 98% at 95 °C, 410–480 bar, and within 20–25 min. These results are very comparable to the results obtained by Hunt et al.^13^ Marín et al. performed a follow-up study where they determined and optimized the extraction of diisoheptyl phthalate (DIHP), DINP, DIDP, and DOP in PVC with scCO_2_ with the focus on surface/volume ratio of the samples and different temperature, pressures, and extraction times.^15^ They obtained efficiencies higher than 90% at temperatures ranging from 90 to 100 °C and pressures above 340 bar for 30 min. Moreover, the greater the S/V ratio and thus larger the surface area, the more rapid and complete the extraction. Guerra et al. conducted extraction and analysis of other plasticizers in PVC such as citrate and benzoate.^16,17^ Extraction efficiencies higher than 99% were achieved at optimal extraction conditions, which were in the range of 390–460 bar, 95–100 °C, and 20–30 min extraction time.

All of these extraction efficiencies of plasticizers are extremely high. Therefore, it might be interesting to use scCO_2_ extraction not only for the quantification but also for the removal of plasticizers from (used flexibly) PVC. An extraction process consists of a series of sequential steps: first, the substrate is impregnated by diffusion of scCO_2_ into the matrix/particle/material; second, the adsorption of CO_2_ on the surface of the matrix forms an external liquid film around the material; and third, the transport of solute from the core of the particle to its surface. Finally, there is transport of the solute molecules by the scCO_2_ flow. The solubility of the solutes in scCO_2_ and the mass transfer out of the matrix are often the rate-determining steps whereby the mass transfer rate is controlled by molecular diffusion and convection.^18,19^ Ideally, a theoretical model based on conservation balances and transport mechanisms, i.e., (differential) mass balances, would be developed. Several differential solute mass balances are required for these models such as the solute in the SCF, solid phase (eventually in the liquid phase when extra solvent is added into the process), and a solute equilibrium desorption that describes the interactions between solute and the solid matrix. These models are very suitable for the development and design of industrial-scale extraction processes. However, in order to be able to design an extraction process, several process parameters, such as mass transfer characteristics, phase equilibria of the materials, and surface area, are required. Unfortunately, the required information is not available due to the lack of research into these topics.

Nevertheless, Sovová developed a simplified approximate model that divides the process into three extraction regions due to the lack of physical process parameters.^20^ The process of SFE consists of three stages: fast extraction, characterized by a constant extraction rate (CER); decreasing extraction, characterized by a falling extraction rate (FER); and slow extraction, completely controlled by diffusion in the solid phase (DC). This model is widely accepted and frequently used for the description of oily substances from plant material.^21−25^ The oily substances are not chemically bound to the plant material but spread throughout the plant and cells. This makes these extraction processes quite similar to the extraction of DOP from PVC.

This study provides an overview of the extraction of DOP, which is frequently used in PVC, from PVC using scCO_2_. The parameters affecting the extraction process are discussed. To investigate the extraction kinetics of this supercritical fluid extraction (SFE), the extraction model established by Sovová was applied.^20^ Moreover, the influence of the extraction process on the properties of PVC will be analyzed in order to discuss the possibility of SFE to industrial waste PVC.

## 2 Experimental Section

### 2.1 Materials and Chemicals

Poly(vinyl chloride) (PVC) with an average *M*_w_ ∼ 80,000 and average *M*_n_ ∼ 47,000, and dioctyl phthalate DOP, ≥99.5%, boiling point 384 °C, vapor pressure 0.165 Pa at 30 °C were obtained from Sigma-Aldrich (Darmstadt, Germany).^26^ Tetrahydrofuran stabilized with BHT (THF stabilization/BHT, ≥99.9%) was purchased from Sigma-Aldrich (Darmstadt, Germany). Carbon dioxide (technical grade, 99% pure) was purchased from Westfalen Gassen Nederland BV (Deventer). For the ^1^H NMR measurements, dimethyl sulfoxide-*d*_6_ (DMSO-*d*_6_, ≥99.9 atom % D, Sigma-Aldrich) was used as a deuterated solvent.

### 2.2 Preparation of PVC/DOP Blends

PVC/DOP blends with a formulation of ∼33 wt % of DOP were made by adding 10 g of DOP to 200 mL of tetrahydrofuran, after which the mixture was stirred for 10 min before adding 20 g of PVC. Then, the mixture was heated to 35 °C and stirred until all PVC was dissolved. Afterward, the mixture was poured into a large Petri dish (diameter 135 mm) and covered with aluminum foil in which a few holes were made. The film was left at room temperature for 10 days, after which it was removed from the dish. The large films were cut into small uniform round disks with a diameter of ∼5 mm and a thickness of 1–1.5 mm.

### 2.3 Instrumental

Continuous extraction experiments were carried out by using an SFE 100 mL extraction system (Extratex S.F.I., Neuves Maisons, France), which consists of an SFE 100 mL stainless steel 316 L extraction vessel that is placed in an oven, a 300 mL stainless steel cyclonic separator, a high-pressure solvent pump, and an integrated controller automatically controlled with a frequency drive. The setup is designed for operations up to 700 bar, temperatures up to 150 °C, and flow rates up to 100 g/min of CO_2_.

**Figure 1:**
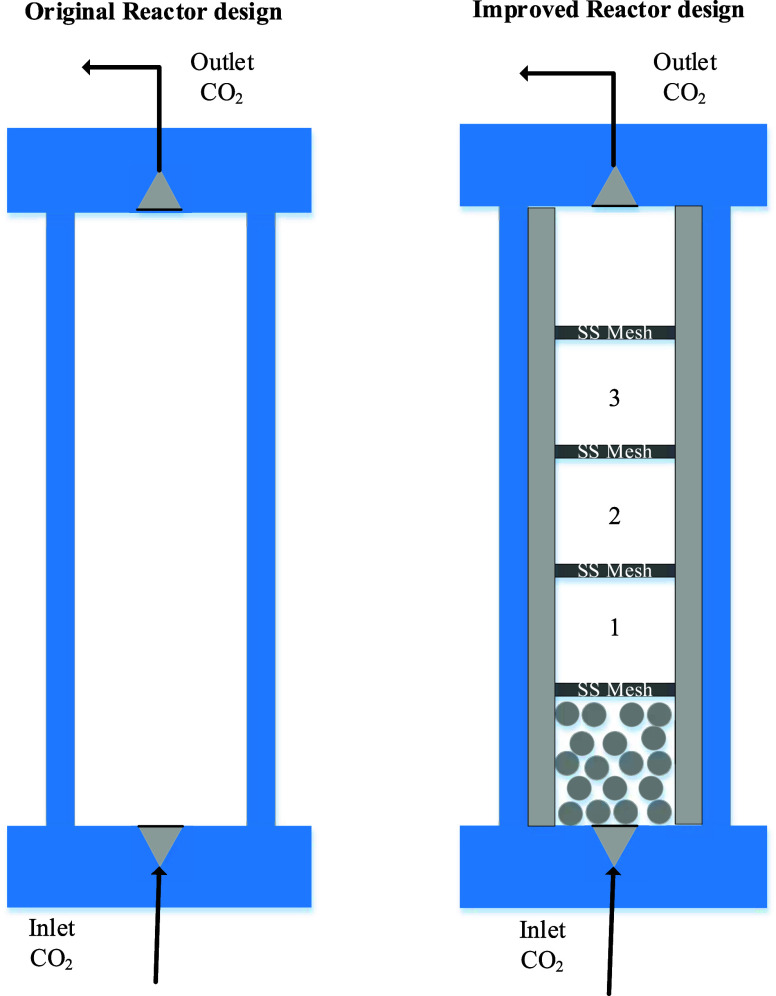
Original (left) and improved extractor (right) for extraction.

However, some significant adjustments have been made to the original setup from the supplier. Originally, the extractor was put in a holder with an angle of 20°, which led to a not perfectly vertical extractor position. This results in a not well-defined flow profile, which can lead to erroneous results. Therefore, the holder was replaced in order to ensure a perpendicularly positioned extractor. Unfortunately, the inlet was not a cone-shaped inlet and also not at the outlet. This causes a jet flow at the inlet of the extractor and eddies in the corners of the entrance and the exit, respectively. Consequently, the design of the extractor was improved to create a laminar or turbulent flow profile inside the extractor. In order to guarantee one of these profiles, an inner tube was created that consisted of five separate parts. The lower part of the pipe had a height of 4 cm and was filled with little RVS balls with a diameter range from 1 to 2 mm. The other 3 parts that go on top of the first part will all three be filled with ∼7.6 g of PVC disks have a height of 3 cm and an iron grid to keep the PVC disks in place. The fifth part also has a height of 4 cm and was left empty. The inner diameters of these five parts are 2.5 cm. A schematic impression is given in Figure 1.

**Figure 2:**
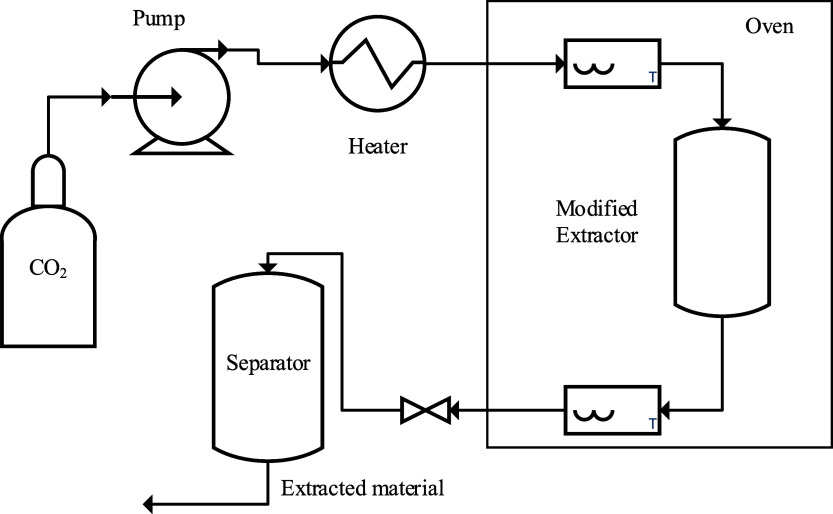
Schematic photo of the improved setup.

Moreover, an extra thermocouple was installed in the inlet of the extractor. In the original setup, there is only one thermocouple, which is placed after the outlet of the extractor. In addition, there is a coil in the oven for the extractor entrance. However, it turned out there is far too little coil surface area to heat up the stream of CO_2_ with only this coil to reach the desired oven temperature. The result is that at an oven temperature of 50 °C, and therefore the extractor temperature and its content, the temperature of the incoming CO_2_ flow is well below that of 50 °C. It turned out that at a flow of 30 g/min CO_2_, the actual incoming CO_2_ was only 43 °C. The difference in temperature will be larger when performing experiments at higher flow rates or higher oven temperatures. In order to overcome this disturbing issue, an extra heating capacity was installed just before the oven. This heating element consists of a heating wire of 150 kW wrapped on an RVS coil. Next to this, all of the piping after the oven was insulated to minimize heat loss and clogging of the system. Moreover, the pipe between the back-pressure valve and separator was also equipped with a heating lint. The solute is collected at the separator and weighed at several time intervals. Figure 2 presents a schematic of the new setup.

### 2.4 Analysis of PVC

For the characterization of the PVC before and after scCO_2_ extraction, several analytical techniques were performed.

#### 2.4.1 ^1^H NMR

Determination of the DOP content in the residues was performed using proton nuclear magnetic resonance (^1^H NMR) spectroscopy for continuous extraction experiments. For ^1^H NMR analysis, the spectra were obtained by using an Agilent 400MR spectrometer and using warm dimethyl sulfoxide-*d*_6_ as a deuterated solvent. Three ^1^H NMR analyses were performed for every sample and the average was taken. See the Supporting Information for more details about the calculation of DOP in PVC. The final efficiency was calculated as follows:
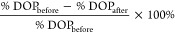
1

#### 2.4.2 Thermogravimetric Analyzer (TGA)

The decomposition temperatures were determined by thermogravimetric analysis on a PerkinElmer TGA 4000. Under a nitrogen atmosphere, samples were heated from 25 to 600 °C at 20 °C/min. The data were processed by using Pyris series TGA 4000 software.

#### 2.4.3 Differential Scanning Calorimetry (DSC)

DSC analysis of the polymer samples was performed on a TA Instruments Discovery DSC 25 equipped with a cooler and autosampler. Samples were prepared using a Tzero aluminum pan and were analyzed via a heat–cool–heat cycle. First, the sample was heated from 45 to 150 °C at a 20 °C/min heating rate, cooled down from 150 to −80 °C at a 20 °C/min cooling rate, and finally heated again from −80 to 150 °C at a 20 °C/min heating rate. The glass transition temperature, *T*_g_, of the PVC was detected, measured in the second heating rate, and processed by the software Trios.

#### 2.4.4 Shear Rheometry

A Discovery Hybrid Rheometer DHR-2 (TA Instruments) equipped with a force rebalance transducer (FRT) was used for the rheological experiments. Stainless steel parallel plates of 8 mm diameter were used for all of the experiments. The temperature was controlled via a convection oven flushed with nitrogen gas to minimize sample degradation. Beforehand, the samples were shaped into disks by means of a hot press at 160 °C and 150 bar and then slowly cooled to room temperature under pressure. The following rheological protocol was adopted and used for the measurement characterizations at 160 °C:

(i) dynamic strain amplitude sweep at ω = 100 rad/s to detect the linear viscoelastic regime
(ii) dynamic frequency sweep at strain amplitudes in the range 0.5–3% and frequency range between 100 and 0.1 rad/s.

When switching samples, a dynamic time sweep in the linear regime was performed in order to ensure stationary conditions, before performing (i) and (ii).

#### 2.4.5 Tensile Test

The mechanical properties of the PVC were measured by using a Tinius Olsen H2SKT set with a pulling speed of 10 mm/min. Samples were prepared by hot pressing at 160 °C and 150 bar in a dumbbell mold with a thickness of approximately 1 or 1.5 mm, gauge width of 5 mm, and gauge length of approximately 12 mm. A temperature of 160 °C was chosen in order to prevent the decomposition of the PVC.

#### 2.4.6 Scanning Electron Microscopy (SEM)

The cross-sectional morphologies of the PVC samples were imaged using a Nova NanoSEM650 operated with an acceleration voltage of 15 kV and a working distance of 6.2 and 6.5 mm. The samples were cryo-fractured in liquid nitrogen and coated with 10 nm of gold before imaging to avoid charging effects. SEM experiments were conducted at room temperature.

#### 2.4.7 Gel Permeation Chromatography (GPC)

Extracted PVC samples at 500 bar and 110 °C underwent GPC analysis utilizing an Agilent model 1200 series, employing 3× PSS GRAM analytical linear columns (300 × 8 mm^2^, 10 μm). The analysis was conducted at a flow rate of 1 mL/min and 50 °C, with an injection volume of 20 μL. THF served as the eluent. *M*_n_ and *M*_w_ values were derived from refractive index chromatograms using polystyrene standards with toluene as the reference peak. Only this PVC sample was tested to investigate the effect of the most extreme extraction conditions on the molecular weight.

## 3 Sovová Model

The extraction process of DOP from soft PVC can be divided into three stages according to Sovová (1994).^20^ The first time period, which is called the constant extraction rate (CER), describes the extraction of easily exposed solutes that occurs through convective mass transfer in the fluid phase. The extraction rate is the highest in this stage. In the second time period, the falling extraction rate (FER), the partial solutes on the surface become depleted. The solute, DOP, in the solid phase, PVC, is extracted through a combination of convection and diffusion, and the extraction rate is slowing down. The final period is diffusion-controlled mass transfer in the solid phase. This period is called diffusion-controlled rate (DCR). To initiate the change from one extraction period into the other, the solute fraction is divided into the part located on the surface of the solid particle that is easily accessible (x_p_) and the other part located inside the particle that is hardly accessible (x_k_). The model assumes that the bed of the solid particles is homogeneous regarding the particle size and location of solute. Moreover, it neglects axial dispersion and diffusion in the solid phase, and the extraction process is assumed to be isothermal and isobaric. In addition, accumulation in the fluid phase is neglected.

For the detailed derivation of the model referred to in Sovová, the extracted solute as a function of time yields the following equations:

For the constant extraction rate, CER: 0 ≤ *t* ≤ *t*_CER_
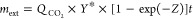
2

For the falling extraction rate, FER period: *t*_CER_ < *t* ≤ *t*_FER_
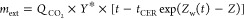
3

For the diffusion-controlled rate, DCR period; *t* > *t*_FER_
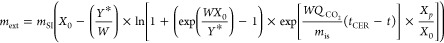
4where *m*_ext_ is the extracted mass from the starting material (kg), the scCO_2_ mass flow is given by *Q*_CO_2__ (kg/s), Y* is the solubility of the solute in the fluid (kg/kg), and t is the extraction time (s). The total starting material is given by *m*_SI_ (kg), *X*_0_ is the mass fraction of total solute in the initial material (kg/kg), *X*_p_ is the mass fraction of the easily accessible solute (kg/kg), and *X*_k_ is the mass fraction of intracellular solute from intact cells (kg/kg). *Z*, *Z*_w_, and *W* are dimensionless parameters given by
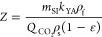
5
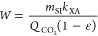
6

The parameters *Z* and *W* are directly proportional to the mass transfer coefficient in the solvent, *k*_YA_ (s^–1^), and the mass transfer coefficient in the solid phase, *k*_XA_ (s^–1^), and are inversely proportional to the flow rate of CO_2_. Moreover, the densities of the fluid and solid are given as ρ_f_ and ρ_s_ (kg/m^3^) and ε is the porosity of the packed bed. *Z*_w_ is shown below:
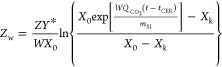
7

The two time periods, *t*_CER_ and *t*_FER_, indicating a new extraction phase are given as
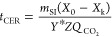
8
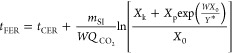
9where *t*_CER_ is the extraction time at the end of the CER period (s) and *t*_FER_ is the extraction time at the beginning of the diffusional period.

## 4 Results and Discussion

The influence of temperature and pressure on the extraction efficiency will be discussed in this section as well as the Sovová fitting parameters. Moreover, the impact of the extraction process on the PVC characteristics is investigated and compared to pure PVC before scCO_2_ extraction. Three different approaches were used to determine the DOP content during and after the experiments. For the first approach, three samples are taken before and after extraction from the PVC disks and prepared for ^1^H NMR to obtain the DOP concentration. These ^1^H NMR results are considered to be the most accurate. The second approach, the gravimetric one, involves weighing the mass of the PVC before and after the experiments. It must be noted that it can take a few days before the weight stabilizes because the CO_2_ is still being dissolved in the polymer. During the extraction, DOP was collected at the separator and the amount was weighed at certain time intervals in order to monitor the removal of DOP versus the time. However, there is some loss of DOP content while measuring the mass collected at the separator. Due to the low vapor pressure of DOP, it can safely be assumed that no DOP was evaporated. It might be possible that some of the DOP remains either in the piping of the setup or in the circulating scCO_2_ at a kind of equilibrium loading. Therefore, it was decided that a correction should be added to the collected DOP to cover this loss. For more details, see the Supporting Information.

### 4.1 Extraction Kinetics and Model Fitting on the Experimental Data of This Study

First of all, in order to check the validity of the developed mathematical code (MATLAB) for the Sovová model, the code was tested with the results presented by Jingfu et al.^21^ The fitting parameters do show an excellent fit with the experimental data and their parameters, as can be seen in Table S1.

The operating conditions and properties of scCO_2_ and PVC are outlined in Table 1. This table includes the chemical and physical properties of the parameters and kinetics equations, as well as the model parameters derived from fitting the present experimental data. Additionally, the calculated time periods of the CER and FER stages are presented in this table. The mass fraction of total solute in the initial material (kg/kg), *X*_0_, was divided into two parts: reversible and irreversible. Not the initial mass fraction as measured by ^1^H NMR before the experiments was used, but the maximal extracted fraction to obtain a better fit, defined as the difference in mass fraction initially and the remaining irreversible mass fraction at the end of the experiment. Moreover, it is assumed that the scCO_2_ density does not change because of the very low amount of DOP.

**Table 1:** Operating Conditions and Fitted Parameters of the Sovová Model for scCO_2_ Extraction of Plasticized PVC

*T* (°C)	110				90		75
*P* (bar)	200	300	500	500	300	500	500
*M*_in_ (g)	23.12	22.95	23.14	11.49	23.05	22.96	23.02
*Q* (g/s)	0.5	0.5	0.5	0.5	0.5	0.5	0.5
ρ_CO_2__ (g/cm^3^)^a^	0.437	0.622	0.791	0.791	0.703	0.847	0.890
ρ_solid_ (g/cm^3^)^b^	1.359	1.365	1.376	1.376	1.378	1.388	1.395
ε	0.616	0.616	0.616	0.307	0.616	0.616	0.616
*Y* (g_DOP_/kg_CO_2__)^c^	1.600	2.973	3.893	3.893	2.053	1.867	1.150
*X*_0_	0.319	0.317	0.336	0.33	0.309	0.273	0.255
*k*_XA_ (×10^–5^)	1.60	2.08	2.54	4.27	1.53	4.22	2.74
*k*_YA_ (×10^–2^)	5.19	2.20	1.83	1.44	3.14	2.72	1.69
*X*_k_	0.203	0.211	0.213	0.190	0.207	0.211	0.190
*Z*	2.01	1.115	1.27	0.275	1.92	1.99	1.29
*W*	0.002	0.0025	0.0031	0.0014	0.0018	0.0050	0.044
*t*_CER_	27.6	24.4	19.2	49.9	20.0	12.8	26.0
*t*_FER_	90.1	54.0	45.7	64.1	61.9	46.4	67.0

a The scCO_2_ density was from the NIST database.

b The PVC density was obtained from the Sanchez–Lacombe equations of state.^27,28^

c The calculation was performed by calculating the solubility of the first part of the extraction curve.

**Figure 3:**
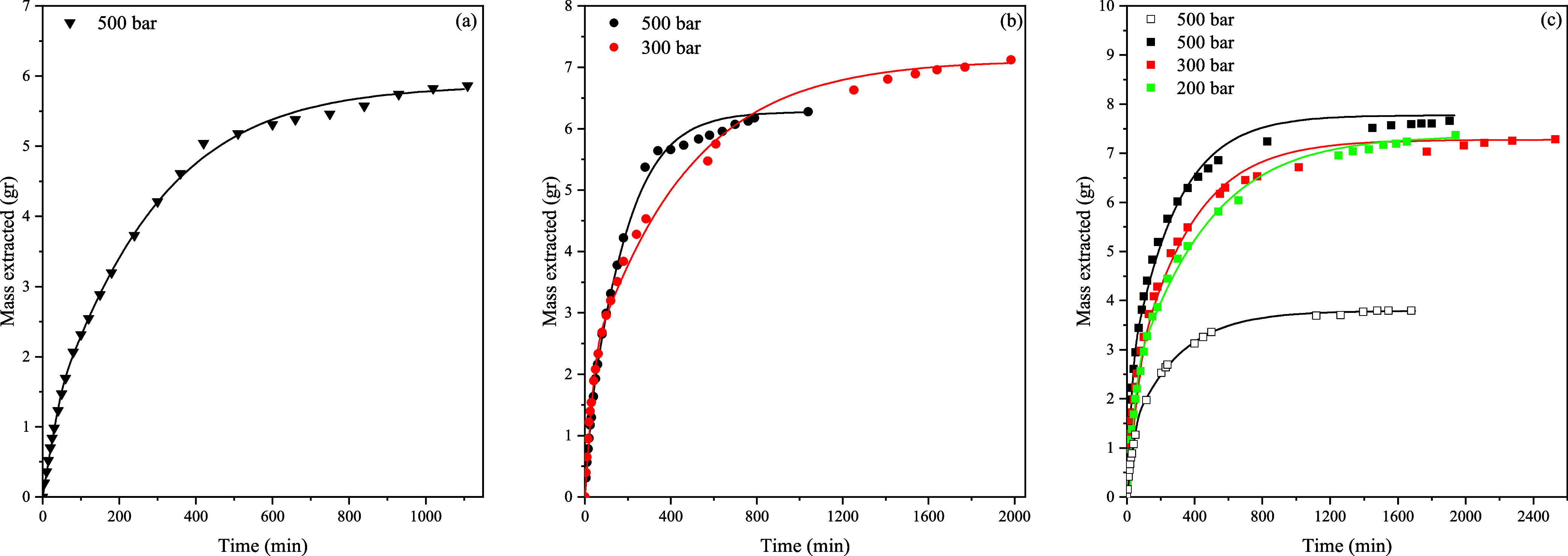
Yield of DOP extraction from PVC at 75 °C (a), 90 °C (b), and 110 °C (c). The lines represent Sovová’s model fitting.

Figure 3 represents the experimental results and predicted overall extraction curves of the Sovová model for the scCO_2_ extraction of DOP from PVC according to ^1^H NMR as explained in Section 2. As expected, for the first extraction period, the extraction rate is the highest due to the easily accessible DOP, whereas the extraction rate decreases with time because the easily accessible DOP has already been removed, the remaining DOP is more difficult to access, and the mass transfer is diffusion-controlled. This is also confirmed by comparing the mass transfer parameter of the solvent phase, *k*_YA_, with the mass transfer parameter in the solid phase, *k*_XA_, which is about 3 orders of magnitude lower.

*X*_k_ is the mass fraction of intracellular solute from intact cells (kg/kg) and is almost similar for all of the tested batches, namely, around 0.2, which indicates that the synthesized batches were very reproducible. *k*_XA_ increased with pressure, probably due to an increase of swelling of the PVC, thus making the DOP more accessible for the scCO_2_. On the other hand, *k*_YA_ decreased with pressure at 110 °C. According to eq 5, *k*_YA_ is inversely proportional to the scCO_2_ density and therefore will decrease with an increase of density caused by an increase of pressure. These observations were also found by Rodríguez-Seoane et al.^22^

At 110 °C and 500 bar, an experiment was carried out with about 50% of the mass (and volume) of PVC in the extractor. From the results of these experiments, it seems justified to conclude that the extraction conversion as a function of time is independent of the amount of PVC present in the extractor. The obtained fitting parameters for the Sovová-model differ substantially compared with the parameters derived from the experiment with the double amount of PVC present in the extractor. It must be noted, however, that the Sovová-model does not solve simultaneously the mass transfer steps intraparticle and external mass transfer as a function of time and position in the extraction unit. Moreover, the solubility of DOP in scCO_2_ is also not required explicitly and was calculated from the first part of the extraction curve. For an extraction model based on first principles, intraparticle diffusion in series with external mass transfer for both experiments are identical, and mass transfer parameters will be derived. Therefore, the calculated *k*_YA_ and *k*_XA_ are a kind of pseudo-mass transfer parameter only valid for the Sovová model within the boundaries of the experimental conditions and are absolutely not identical to the actual mass transfer coefficients used for dimensionless mass transfer correlations.

However, the broken and intact cell theory of Sovová as originally developed for the extraction of solutes from bio-based resources is very well able to describe the present experimental data and can also be applied to a wider range of chemical processes.^20^

### 4.2 Influence of Temperature and Pressure

**Figure 4:**
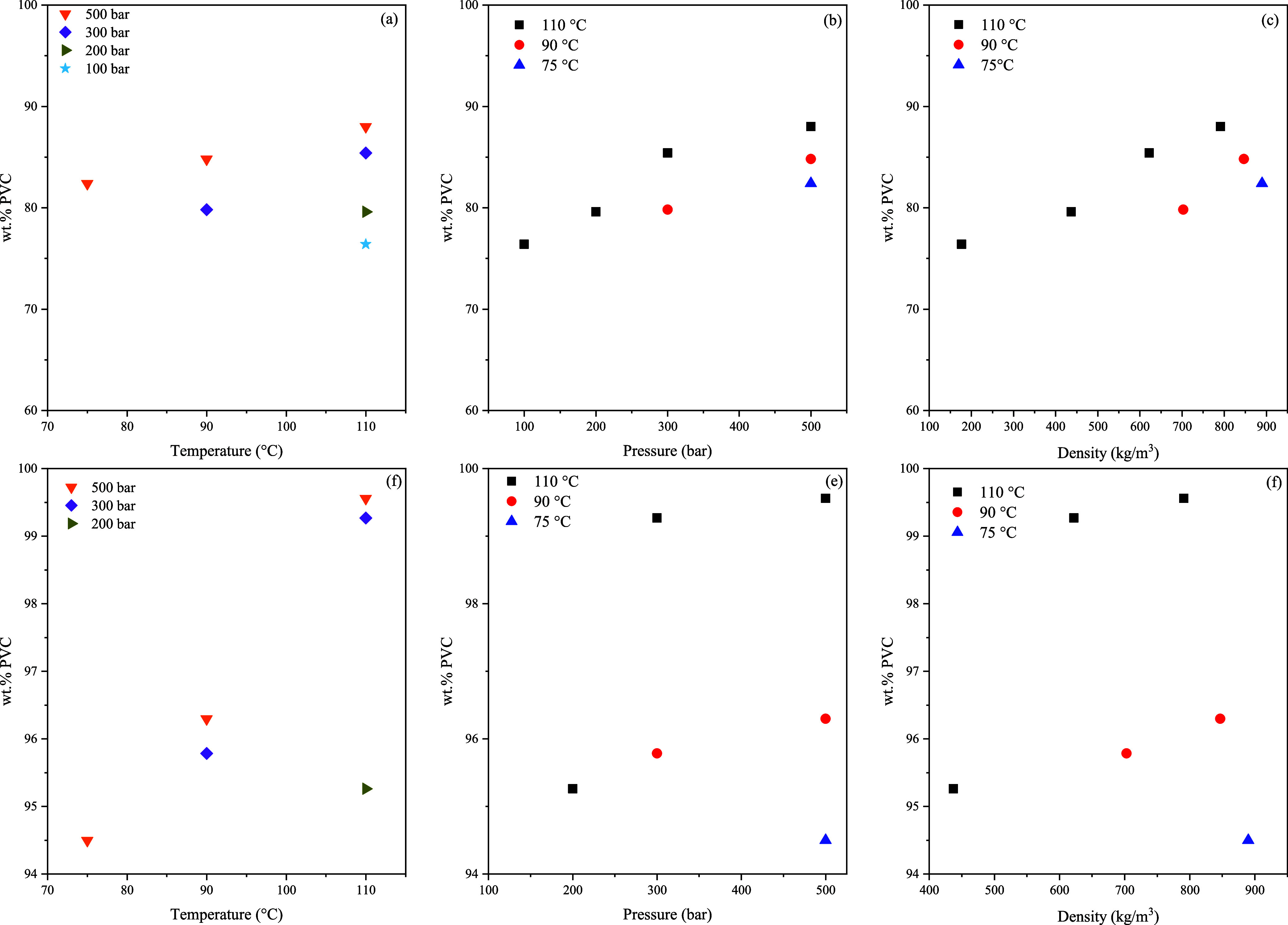
Extraction results, represented by the PVC purity, according to ^1^H NMR after 240 min for different temperatures (a), pressures (b), and CO_2_ densities (c), and at the end of the experiments for different temperatures (d), pressures (e), and CO_2_ densities (f).

Experiments were carried out at different temperatures and pressures in order to investigate the influence of these two parameters on the extraction efficiency of DOP from PVC with scCO_2_. The pressures studied were 100, 200, 300, and 500 bar, and three different temperatures, 75, 90, and 110 °C, were tested. The sample compositions are between 30 and 35 wt % DOP and 65–70 wt % PVC before extraction. The PVC sample composition after 240 min of extraction is given in Figure 4a–c. In Figure 4d–f, the maximum attainable PVC compositions, reached after more than 1000 min, are presented and determined with ^1^H NMR.

**Figure 5:**
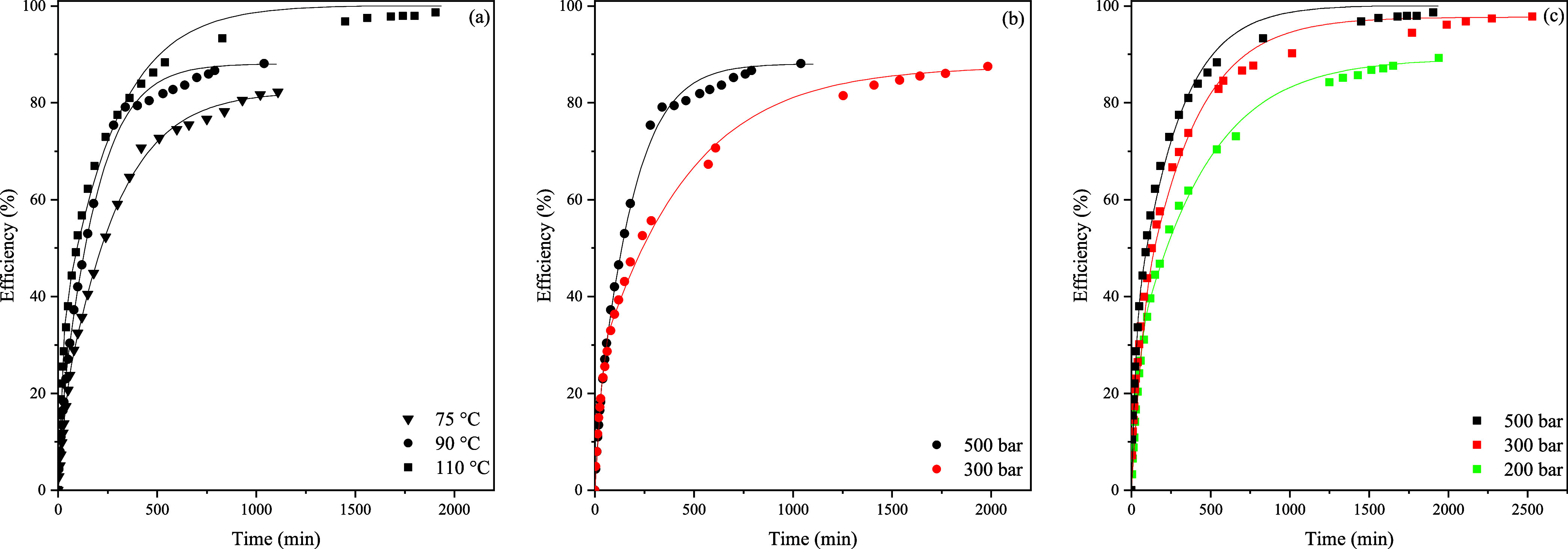
Extraction efficiencies for three different temperatures (75, 90, 110 °C) at 500 bar (a). Extraction efficiencies were 300 and 500 bar at 90 °C (b). Extraction efficiencies for 200, 300, and 500 bar at 110 °C (c). The lines represent Sovová’s model fitting.

Figure 4a–c shows the results of the first 240 min of extraction where more than 50% of the DOP has been removed. The run at 100 bar was stopped after 240 min, as it was not possible to collect DOP at the separator and therefore keep track of the extraction rate; see the Supporting Information for more details. According to Figure 4d–f, PVC samples of more than 94 wt % PVC were achieved and almost complete pure PVC at extraction conditions of 110 °C for 300 and 500 bar. In Figure 4b,e, it can be seen that higher temperatures are favorable at lower pressures for the removal of DOP. It is interesting to note that it is not necessary to apply the high-pressure levels as is shown in Figure 4c,f, where the largest CO_2_ density did not result in the fastest and highest extraction efficiency. The extraction curves of the efficiencies are plotted in Figure 5.

**Figure 6:**
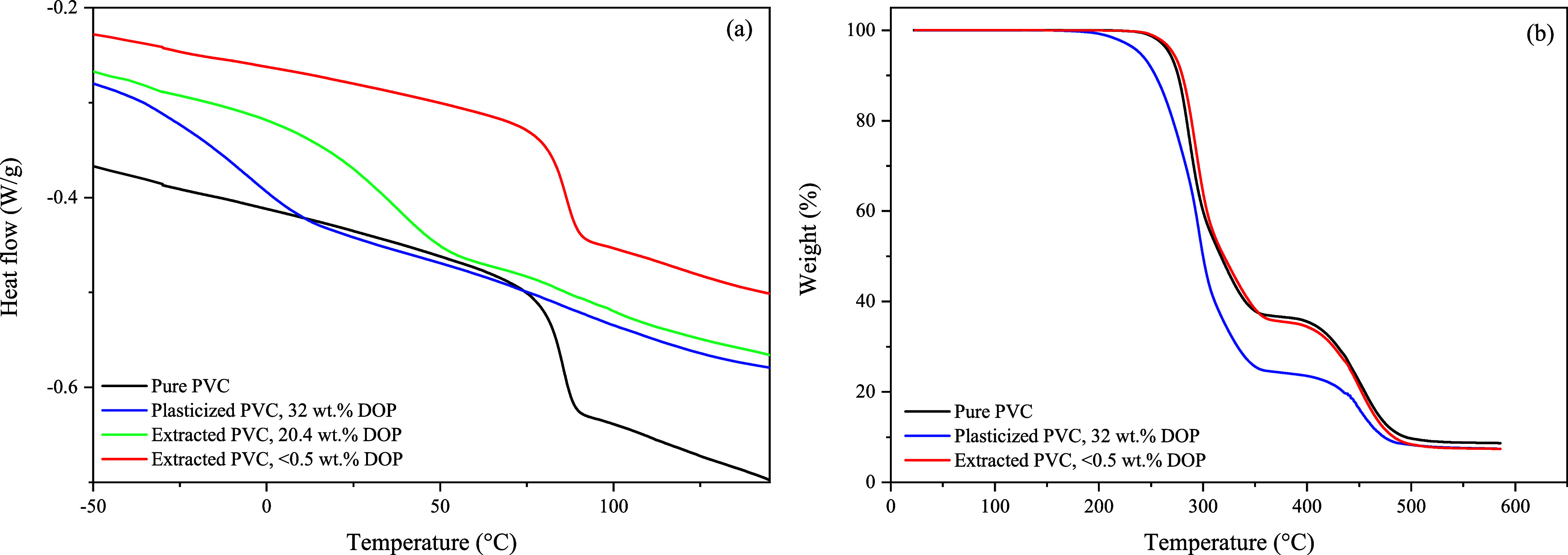
Results of DSC analysis of pure and (semi)extracted PVC (a). The second heating cycle is presented. Results of TGA of pure and (semi)-extracted PVC (b).

According to Figure 5, increasing the pressure as well as the temperature resulted in an increase in the extraction efficiency and a higher extraction rate. During the first extraction period, the extraction rate is the highest, which results in high efficiencies within a short time span. The extraction efficiency will level off, and subsequently, a plateau is reached. This plateau was also observed by Hunt et al. and Marin et al.^13−15^ The influence of pressure is shown in Figure 5b,c. Increasing the pressure has a positive influence on the extraction efficiency. This is probably caused by the CO_2_ sorption into the PVC enhancing the diffusion rate of the DOP through the PVC matrix even though the dense gas pressure is inversely proportional to diffusivity. According to Muth et al., the highest values of the diffusivities were found at the temperature/pressure maximum, which explains the highest efficiencies at 110 °C and 500 bar.^29^ Moreover, most likely the solubility of DOP increases with pressure.^30^ At higher temperatures, the density of CO_2_ decreases, resulting in a lower solvation power. On the other hand, the diffusion will be enhanced and the PVC matrix softens at higher temperatures leading to an increase of extraction efficiency. The latter can be seen in the *T*_g_ change of the PVC during the extraction process as well as the TGA results and is given in Figure 6.

As expected, *T*_g_ increases with a decrease of the DOP content. Küppers et al. also mentioned that for an extraction process, a variation in the temperature of extraction could be favorable when it is close to the *T*_g_.^31^ According to Marin et al., this would not be applicable for phthalate extraction from PVC because plasticized PVC has a *T*_g_ starting at −25 °C, as can be seen in Figure 6.^14^ Furthermore, CO_2_ has a plasticizing effect on the PVC reducing the *T*_g_ as well during the extraction.^29,32^ Therefore, the influence of diffusion and solubility enhanced the extraction process. Hence, for complete removal of DOP from PVC, the usage of temperatures higher than the *T*_g_ of pure PVC will lead to shorter extraction times, as well as higher maximum attainable extraction efficiencies.

**Figure 7:**
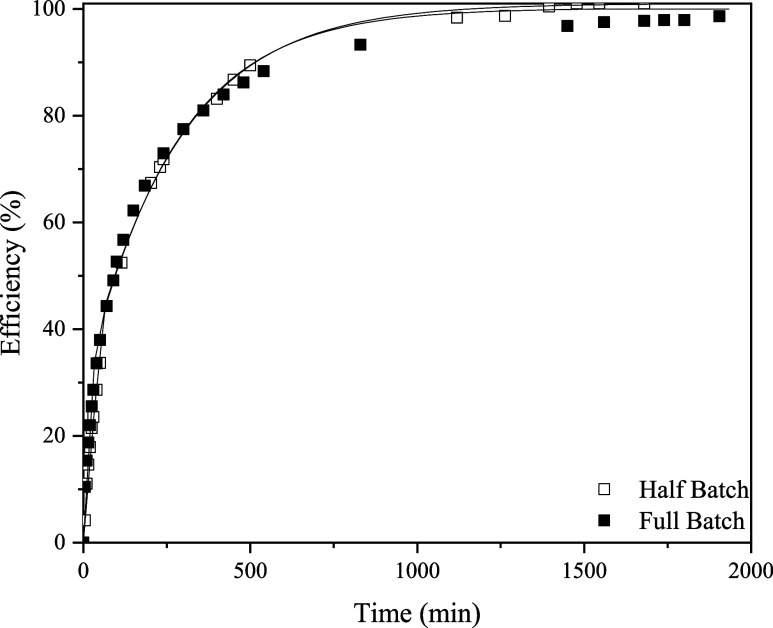
Extraction efficiencies of two batch sizes measured at 500 bar and 110 °C. The lines represent Sovová’s model fitting.

Figure 7 shows the extraction efficiencies of two batch sizes, 11.49 and 23.14 g, at 500 bar and 110 °C. As stated before, it can be concluded that the extraction of DOP from PVC can be regarded as a first-order process. As mentioned previously, the Sovová mass transfer parameters are not mass transfer coefficients; otherwise, they would have given the same results.

It must be noted that the work performed by Hunt et al. and Marín et al. was carried out at a significantly smaller scale compared to the present work.^13−15^ Moreover, they used an open system meaning that during extraction, only fresh, unloaded CO_2_ is added into the extractor cell, whereas the present experiments were performed with a closed-loop system of CO_2_. Therefore, the extraction times of experimental work cannot be directly compared.

Overall, the removal of DOP from PVC is extremely high and in line with the findings of Hunt et al. and Marín et al.^13−15^ Increasing the scCO_2_ flow rate or continuously adding fresh CO_2_ together with a purge stream of loaded CO_2_ might reduce the overall extraction time as well as improve the final extraction efficiency at more mild conditions due to the occurrence of an equilibrium of DOP and CO_2_ that might form during the experiments.

### 4.3 Effects on Polymer Structure

First, the PVC extracted at 500 bar and 110 °C was analyzed by GPC, and it appeared that there was no change in the molecular weight of the PVC compared to pure PVC, as can be seen in the Supporting Information. However, during the extraction experiments, PVC will be saturated with CO_2_. This absorption of CO_2_ modifies the polymer by swelling the matrix and increasing its free volume. Moreover, before extraction, the plasticized PVC disks were flat, transparent, and flexible while the PVC disks after the DOP extraction were stiff, spherical, and opaque. The increase of stiffness is caused by the removal of the plasticizer; however, the change from transparent to opaque can be caused due to the generation of a microcellular foam with cells with dimensions in the size to be able to scatter visible light according to Muth et al., which is also confirmed by the changing shape of the disks.^29^ Furthermore, before the PVC properties were investigated, the samples treated with scCO_2_ were evaluated at least 2 weeks after the extraction due to the slow dissolution of CO_2_ from the PVC matrix.^29,32^ Rheology, tensile tests, and SEM were performed in order to analyze the influence of CO_2_ on the PVC characteristics. All of the tested samples were pressed at 160 °C and 150 bar before further analysis.

#### 4.3.1 Rheological Results

**Figure 8:**
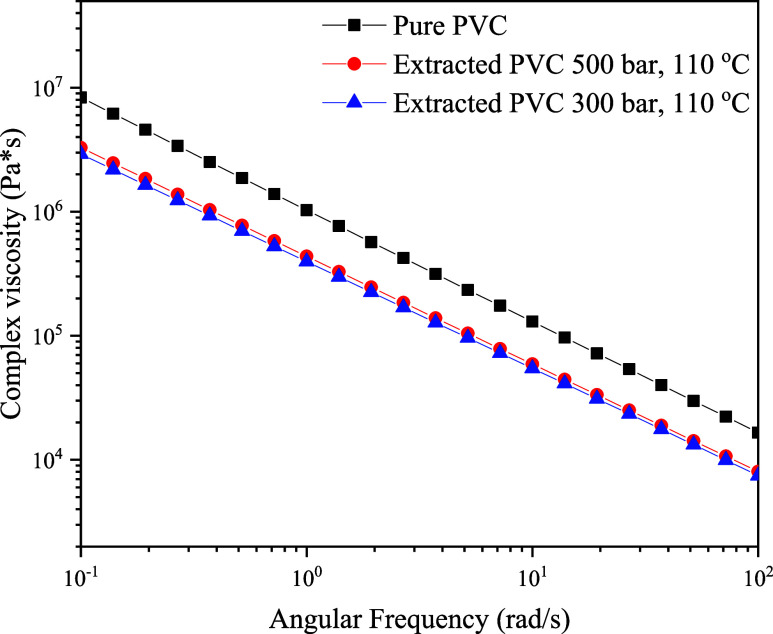
Frequency spectra in terms of complex viscosity as a function of oscillation frequency for three different PVC samples: Pure nonplasticized PVC (black square), PVC extracted for 1906 min at 500 bar and 110 °C with <0.5 wt % DOP (red circle), and PVC extracted for 2530 min at 300 bar and 110 °C with <0.7 wt % DOP (blue triangle).

For rheology, pure PVC and two samples of extracted PVC were tested. These two extracted PVC samples were chosen because they almost have no DOP in the matrix present. The complex viscosity, obtained from the dynamic frequency sweep tests, was used as the main rheological function for comparison. In Figure 8, the complex viscosity of the tested samples is plotted against the angular frequency at 160 °C.

As can be seen in Figure 8, the complex viscosities of the treated PVC samples are significantly lower compared to the pure PVC sample. The two extracted PVC samples differ relatively only slightly from each other, which suggests that the influence of pressure is lower compared to the influence of CO_2_ per se.

#### 4.3.2 Tensile Test

**Figure 9:**
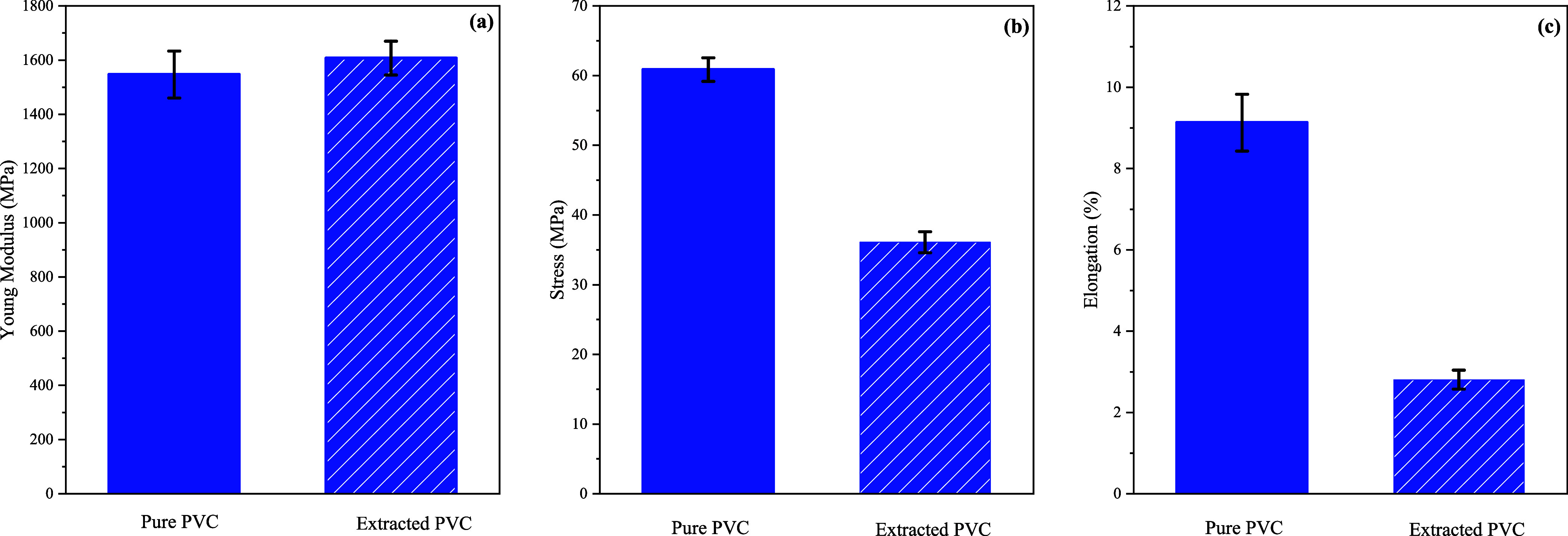
Young’s modulus (a), stress (b), and elongation at break (c) for pure PVC and extracted PVC for 1906 min at 500 bar and 110 °C with <0.5 wt % DOP, both pressed at 160 °C for 10 min.

To further analyze the influence of the CO_2_ extraction process on the properties of PVC stress–strain, tensile tests at room temperature were performed. The three different tensile properties that were tested are stress and elongation at break, and the Young’s modulus, which were obtained from the stress–strain curves. The extracted PVC was almost free of DOP, and the process conditions were 500 bar and 110 °C. This extracted PVC was chosen because it withstands the highest pressure and temperature compared to the other extraction experiments. The results are given in Figure 9.

According to Figure 9a, the Young’s moduli of pure PVC without DOP, and extracted PVC, are almost equal. However, a larger deviation is observed for the stress and elongation at break, as given in Figure 9b,c. Extracted PVC is considerably weaker compared to unprocessed PVC. This observation is in line with the results of Shieh et al., who also found a significant decrease in yield strength after treatment with CO_2_ at a pressure of 207 bar.^32^ On the other hand, Shieh et al. found an increase of elongation after CO_2_ treatment, whereas in this study, a significant decrease has been observed.^32^ Overall, treatments of PVC with scCO_2_ do affect the mechanical properties of PVC leading to a material that is weaker and less flexible.

#### 4.3.3 SEM

The rheology results and the tensile test results showed that the treated PVC has different polymer characteristics than pure PVC. According to the DSC and TGA results, given in Figure 6, there is no change in thermal properties. Additional morphological analysis using SEM was conducted to explore the potential impact of CO_2_ sorption on crystallization or the formation of a microcellular structure in the PVC.^32^

**Figure 10:**
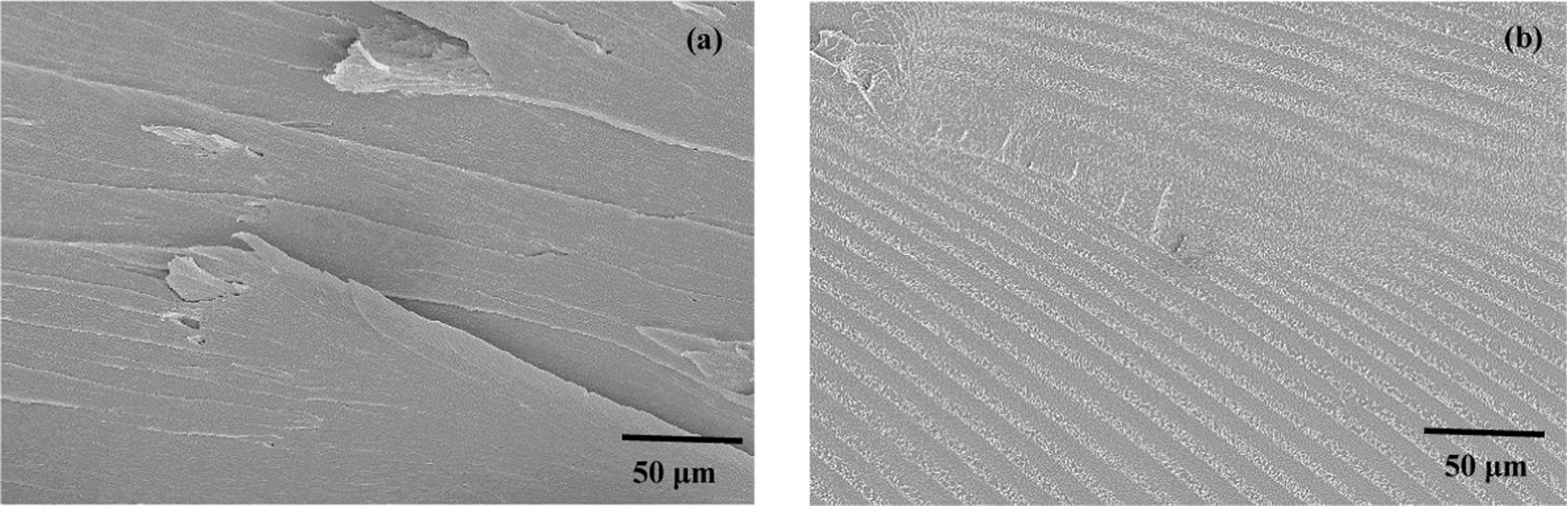
Cross-sectional morphological structures of pure PVC (a) and extracted PVC at 500 bar and 110 °C (b).

**Figure 11:**
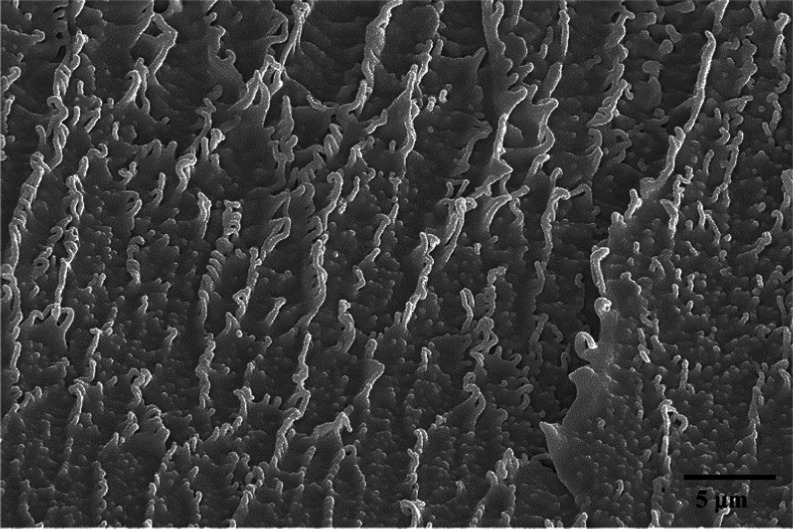
Cross-sectional morphological structures of extracted PVC at 500 bar and 110 °C.

As mentioned previously, the treated PVC disks are opaque and spherically shaped, indicating that foaming has occurred. However, conducting rheology and tensile tests, the treated PVC disks were heated and pressed in disks and dumbbell-like shapes at 160 °C making the samples transparent again. This phenomenon was also reported by Muth et al.^29^ The results of the SEM are listed in Figures 10 and 11.

The SEM results in Figure 10 revealed several differences in the polymer structure of the scCO_2_ extracted PVC compared to regular PVC. The pure PVC showed a uniform and homogeneous structure with an almost smooth surface. On the other hand, the SEM photo of the extracted PVC displayed a different structure with areas covered in small dark and white lines. In Figure 11, an enlarged SEM photo is given of the cross section of the extracted PVC.

The extracted PVC showed a very interesting structure and a nanofibril structure, respectively. The nanostructure is no longer firmly connected, and there are open spaces between the fibrils that probably cause a reduction in stress and elongation. This means that even after hot pressing the extracted PVC, significant changes are observed in the morphology of the extracted PVC. This phenomenon might be attributed to the fact that scCO_2_ acts also as a foaming agent and indeed the visual appearance of the polymer after extraction is the one of a light foam. This is contrary to the findings of Muth et al.^29^ They performed electron microscopy with a magnification of 1 μm, where no cell structures or micro holes were visible. The only different observation they had was the CO_2_-treated PVC appeared brighter because the density of PVC itself is lower. This would also explain the different mechanical properties of our tensile test. Additionally, their X-ray diffraction patterns exhibited many similarities between pure PVC and PVC treated with CO_2_. Therefore, Muth et al. suggested that any alterations in crystallinity attributed to CO_2_ sorption/desorption can be disregarded.^29^ However, our SEM results do display a change in morphological structure after extraction with scCO_2_ despite an additional step of hot pressing.

## 5 Conclusions

Overall, there is a global consensus advocating for the reduction of plastic waste and the enforcement of recycling processes where reduction is not within reach. However, the presence of additives in plastics is a significant obstacle to closed-loop recycling and reuse initiatives within the plastic waste stream. Therefore, it is logical to incorporate a preliminary step in the development of future recycling processes. The present research endeavors to evaluate the efficiency of SFE utilizing scCO_2_ for the removal of plasticizers from PVC.

It is clearly demonstrated that continuous scCO_2_ extraction is a very efficient method for the removal of DOP from PVC. DOP extraction efficiencies of more than 80% were obtained, and almost complete removal of DOP was realized at 110 °C for 300 and 500 bar. This all resulted in PVC samples with more than 94 wt % purity. Moreover, a numerically solved process model based on the Sovová model was developed and successfully applied to describe and simulate the extraction experiments. The influence of scCO_2_ on the PVC has been evaluated and indicated that foaming of the PVC occurs. A reduction in complex viscosity, strength, and elongation was found, but there was no change in the molecular weight of the PVC. The SEM photos exhibited a change in morphology, which showed a nanofibril structure for the extracted PVC sample instead of a homogeneous structure for the pure PVC. This change in morphology can explain the difference in mechanical properties. Therefore, in order to consider the possibility of reusing the treated PVC, further investigation in polymer characterization and analysis is required.

## Supporting Information Available

^1^H NMR DOP content calculations, molecular structures, ^1^H NMR spectra, GPC results, details on extracted mass, and Sovová model literature comparison (PDF)

## Nomenclature

*m*_ext_: extracted mass from the starting material (kg)
*Q*_CO_2__: scCO_2_ mass flow (g/s)
*Y**: solubility of the solute in the fluid (kg/kg)
*t*: extraction time (s)
*M*_in_: total starting material (kg)
ρ_f_: density of the supercritical fluid (g/cm^3^)
ρ_s_: density of the polymer (g/cm^3^)
ε: porosity of the paced bed
*X*_0_: mass fraction of total solute in the initial material (kg/kg)
*X*_p_: mass fraction of easily accessible solute (kg/kg)
*X*_k_: mass fraction of internal styrene (kg/kg)
*k*_XA_: mass transfer coefficients in the solvent phase (s^–1^)
*k*_YA_: mass transfer coefficients in the solid phase (s^–1^)
*Z*: dimensionless parameter
*Z*_w_: dimensionless parameter
*W*: dimensionless parameter
