# Supplementary material for: Recycling PVC with scCO2: From Soft to Rigid PVC
Source: ACS Sustain Chem Eng. 2024 Aug 28;12(42):15398–408. doi: 10.1021/acssuschemeng.4c03743 (PMC11497206; doi:10.1021/acssuschemeng.4c03743)
Supplement: Supplementary file 1 — sc4c03743_si_001.pdf [file sc4c03743_si_001.pdf]

Supporting Information

Recycling PVC with $scCO_2$: from soft to rigid PVC

Frederique A. Versteeg, Diana Bollen and Francesco Picchioni*

Department of Chemical Engineering - Product Technology, University of Groningen, Nijenborgh 4, 9747 AG Groningen, The Netherlands.

*Corresponding Author: f.picchioni@rug.nl

- Number pages:7.
- Number of figures: 4.
- Number of tables: 1.

Contents

^{1}H-NMR DOP calculations S3
Sovová model S4
Mass balance of the $scCO_2$ extraction experiments S5
GPC results S7

^{1}H-NMR DOP calculations

Mass fraction of DOP is calculated in the following way:

1) Peak at δ = 7.72-7.61 is integrated (= A_{DOP})
2) Peak at δ = 4.8-3.85 is integrated (= $A_{DOP+PVC}$)
3) $A_{PVC} = A_{DOP+PVC} - A_{DOP}$
4) Peak DOP represents 4H; peak PVC represents 1H
5) Hence, there is $A_{DOP}/4$ relative mol DOP and $A_{PVC}/1$ Relative mol PVC

$$Mass\ fraction\ DOP = \frac{A_{DOP} * M_{w},DOP}{A_{DOP} * M_{w,\ DOP} + 4 * A_{PVC} * M_{w,\ PVC}} \qquad \text{Eq. (S1)}$$

M_w,DOP = 390.56 g/mol

M_w,PVC = 62.5 g/mol

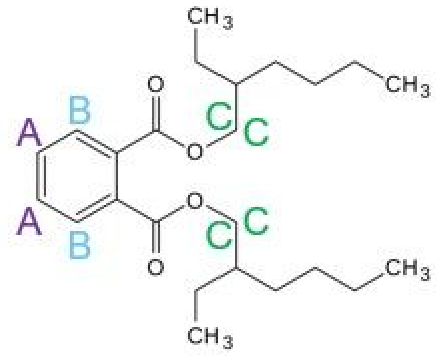

Figure S1. Molecular structure of DOP. A and B represent together 4 hydrogens. C also represents 4 hydrogens.

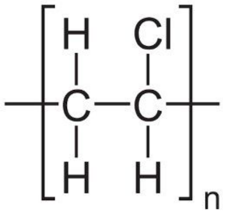

Figure S2. Molecular structure of a monomer of PVC

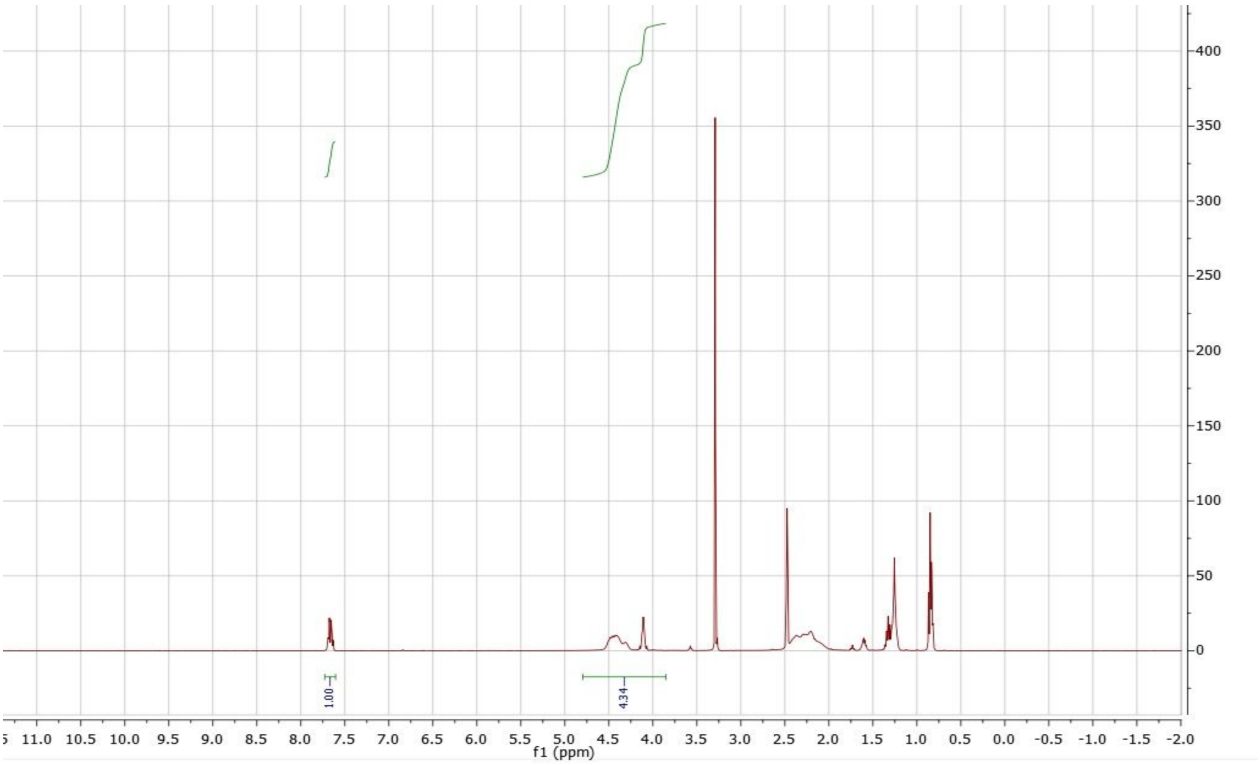

Figure S3. ^{1}H-NMR spectrum of a PVC/DOP strip

Sovová model

The mathematical solution of the Sovová model equations was carried out numerically using MATLAB 2019. Experimental data from Jingfu *et al.* (2022)[21] and their mass transfer parameters were used for tested in order to validation of the developed MATLAB code. The results are given in Table S1. As can be concluded from Table S1, the results derived with the current code is in excellent agreement with the obtained parameter from Jingfu *et al*. (2022)[21].

Table S1. Model parameters

Parameters	Jingfu *et al.* (2022)[21] (Table 1)	Current MATLAB code
	S-GL	***S-GL***
kXa	0.00111	0.00108
kYa	0.00476	0.00479
Xk	0.0349	0.0336
Z	1.903	1.9131
W	0.2519	0.2456
tCER	20.3	20.32
tFER	62.7	62.7
	S-JR	***S-JR***
kXa	0.00105	0.0010112

kYa	0.0401	0.040168
Xk	0.0309	0.029911
Z	1.58	1.5976
W	0.0493	0.0481
tCER	32.7	32.6151
tFER	86.8	86.9526
	R-ZO	***R-ZO***
kXa	0.00082	0.000826
kYa	0.0266	0.02665
Xk	0.0119	0.01216
Z	1.6892	1.6951
W	0.0594	0.0599
tCER	10.3	10.1
tFER	34.1	33.83

Mass balance of the $scCO_2$ extraction experiments

It was found that there is a difference when comparing the ^{1}H-NMR results with the collected grams of DOP at the separator. The results of the ^{1}H-NMR, scale and collected DOP are given in Table S2. Unfortunately, the third option for determining the extraction efficiency via weighing was not an option as, for some experiments, small amounts of the treated PVC pellets could not be retrieved and have been lost. Therefore, the weighed mass loss over time turned out to be not reliable enough to be used for determining the mass balance.

		Before			After				
Conditions	Time (min)	M_{start}	1H-NMR (%)	M_0 DOP (gr)	M_{After}	^{1}H-NMR (%)	^{1}H-NMR (gr)	M_1 DOP$^{\alpha}$ (gr)	Collected DOP (gr)
110 °C. 500 bar	1905	23.14	33.55	7.76	15.32	0.46	0.07 / 7.69	7.82	6.99
110 °C. 500 bar	1680	11.49	32.99	3.79	7.64	0.49	0.03 / 3.76	3.85	3.39
110 °C. 200 bar	1940	23.12	35.7	8.25	15.82	3.85	0.60 / 7.65	7.3	6.58

90 °C. 300 bar	1983	23.05	35.31	8.14	15.98	4.41	0.70 / 7.44	7.07	6.48
110 °C. 100 bar	320	23.12	33.32	7.70	18.75	20.38	3.82 / 3.88	4.37	0.6
110 °C. 300 bar	2530	22.95	32.45	7.45	15.29	0.72	0.11 / 7.34	7.66	6.95
90 °C. 500 bar	1040	22.96	31.03	7.12	15.68	3.7	0.58 / 6.54	7.28	6.1
75 °C. 500 bar	1110	23.02	30.97	7.13	16.04	5.5	0.88 / 6.25	6.98	5.85

Table S2. Removal of DOP measured by ^{1}H-NMR, scale and collected DOP.

$^{\alpha}$Not reliable

In Table S2 it can be seen that, the collected DOP at 110 °C and 100 bar is extremely low compared to the ^{1}H-NMR results. It might be possible that the density of $scCO_2$ at these conditions, 177 kg/m^3, is too low and that the DOP will get extracted out of the PVC pellets but will stay in the reactor because of phase separation instead of being transported to the separator by the $scCO_2$.

Due to the difference in ^{1}H-NMR results and the collected DOP, an additional experiment was conducted at 500 bar and 110 °C but with only half of the usual amount of starting material. As can be concluded, there is not a constant, fixed amount of DOP that remains in the lining of the set-up because under the same conditions, but with a different batch weight, there is also a difference in the amount of missing DOP. It is most likely, that the missing DOP remains dissolved in the circulating $scCO_2$. Therefore, it was decided to add a correction to the collected DOP to cover the loss that is shown in Table S3.

Conditions	M_{total} (gr)	^{1}H-NMR$_0$ (wt.%)	M_0 DOP (gr)	^{1}H-NMR$_{after}$ (wt.%)	Efficiency (%)	Collected DOP (gr)	Correction (%)
110 °C. 500 bar	23.14	33.55	7.76	0.46	98.6	6.99	8.7
110 °C. 500 bar	11.49	32.99	3.79	0.49	98.5	3.39	10.6
110 °C. 300 bar	22.95	32.45	7.45	0.72	97.8	6.95	4.6
110 °C. 200 bar	23.12	35.73	8.25	3.85	89.2	6.58	10.7

90 °C. 500 bar	22.96	31.03	7.12	3.7	88.1	6.1	2.8
90 °C. 300 bar	23.05	35.31	8.14	4.41	87.5	6.48	9
75 °C. 500 bar	23.02	30.97	7.13	5.5	80.2	5.85	0

Table S3. Correction for DOP loss at the separator.

GPC results

Poly(vinyl chloride) (PVC) with an average Mw ~80,000 and average Mn ~47,000 was used for the experimental work. Extracted PVC at 500 bar and 110 °C had an average Mw ~100,000 and average Mn ~46,800 according to the GPC analysis given in Figure S4. Therefore, it can be concluded that high temperature and pressure as well as $scCO_2$ do not affected the molecular weight of the polymer during the extraction process.

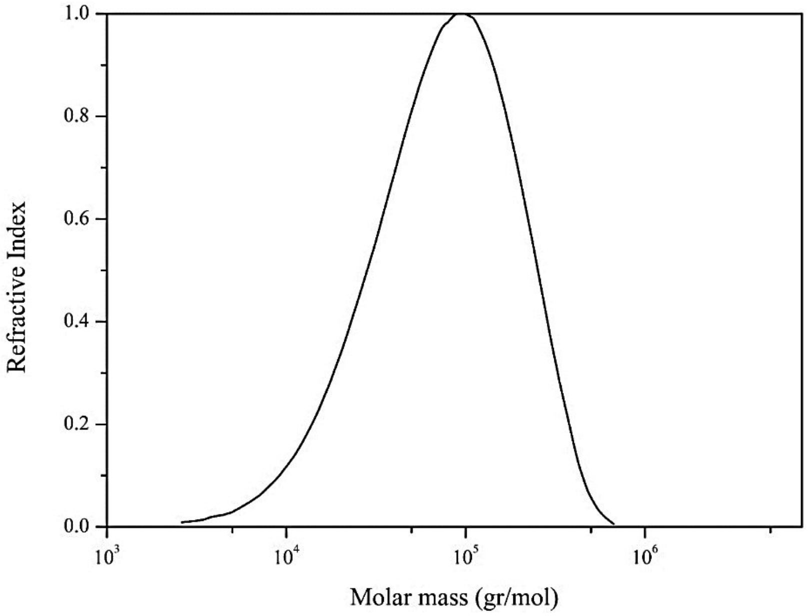

Figure S4. GPC result of Extracted PVC at 500 bar and 110 °C.
